# Supplementary material for: A planetary health perspective on the translation of climate change research into public health policy and practice: A scoping review protocol
Source: PLoS One. 2024 Mar 13;19(3):e0295931. doi: 10.1371/journal.pone.0295931 (PMC10936763; doi:10.1371/journal.pone.0295931)
Supplement: Attachment — Submitted filename: CC review.docx [file pone.0295931.s004.docx]

Page 4

“In 2019 it was estimated that 356, 000 deaths worldwide were link to extreme heat (7),…..”

(i) Change ‘link’ to linked
(ii) Not all extreme heat could be attributed to CC. The authors need to situate this statement appropriately in the context of CC

“In literature, several have been conducted to establish effects on CC………”

(i) Several studies??

“However, CC research knowledge has not been not translated into tangible solution in relation to public health policy and practice”.

(i) Kindly delete ‘not’ before translated

“Integrated knowledge translation (IKT) approach is one strategy that can be utilised to applied”….

(i) To apply??

There are places climate change is shortened as “CC” and in some places written fully. I think this should be consistent throughout the paper

Page 5

‘we have not identified any reviews on decision making in climate change……

(i) We have not identified any review……please delete the ‘s’

Page 8

“Any studies…….”

(i) Rephrase to Any study

Type of participants

This section does not come clear to readers. This is a scoping review so mentioning study participants such as researchers working in the field of climate change etc does come to clear. Maybe authors need to explain this section and the role of those participants in the scoping review.

Are the authors using the PCC framework in this regard? More explanation needed or that whole section could be deleted

Can the authors expand the database to Scopus etc. It appears what has been listed is too limiting.

Again, will the study include only English Language papers? That needs to be stated clearly

Will the entire scoping review be guided by a theoretical framework on CC and Health? If so, this must be stated in the manuscript.

Overall, I think the authors need to read through the manuscript again and correct some basic grammatical errors which could have been avoided.
