# Supplementary material for: A planetary health perspective on the translation of climate change research into public health policy and practice: A scoping review protocol
Source: PLoS One. 2024 Mar 13;19(3):e0295931. doi: 10.1371/journal.pone.0295931 (PMC10936763; doi:10.1371/journal.pone.0295931)
Supplement: Attachment — Submitted filename: PPLos One review 11062023.docx [file pone.0295931.s005.docx]

Thank you for the opportunity to review this manuscript titled: ‘a planetary health perspective on translation of climate change research into public health policy and practice: A scoping review protocol. ‘The authors seek to explore approaches in climate change research knowledge translation, promotion, uptake and public health policy and practice formulation. No doubt the scoping review adds to the existing knowledge on translation of research into policy and practice.

Some comments:

-The significance, rationale and objectives are justified.

-The background – page 4 line 15 ‘Many deaths have been attributed to CC are as a result of increase in temperatures, and *changing patterns of infectious diseases among vulnerable communities* (6). If necessary explain the link between CC and changing patterns of infectious disease among vulnerable groups. Are there other CC related factors responsible for deaths?

-Include some literature on research knowledge translation.

-Need some editing.

-It is commendable that authors will be guided by Arksey and O’Malley methodology. However, this methodology though offers a good foundation, authors should highlight strengths and potential limitations that are inherent in this methodology.

- Identifying relevant studies: Review time is unclear: 2000 onwards (2000-2022) or last two decades. Please clarify.

- The preliminary search strategy using relevant keywords and MeSH terms (Supplementary File 2), I suggest to add AND NOT to Boolean Operators OR and AND.

-Clarify who is conducting the search- the authors, author reviewers, or reviewers. The study will benefit from a librarian?

-Covidence: Please clarify how Covidence will be used in managing this scoping review?
-Collating summarizing, and reporting the results: explain more on qualitative analysis process.
