# Supplementary material for: A planetary health perspective on the translation of climate change research into public health policy and practice: A scoping review protocol
Source: PLoS One. 2024 Mar 13;19(3):e0295931. doi: 10.1371/journal.pone.0295931 (PMC10936763; doi:10.1371/journal.pone.0295931)
Supplement: Attachment — Submitted filename: plos.docx [file pone.0295931.s006.docx]

REVIEWER’S COMMENTS

Pre-testing is necessary for scoping reviews to ensure whether there is existing literature on the research topic. However, the authors failed to produce evidence that their study is feasible.

Knowledge of climate change is one of public health's importance towards achieving the sustainable development goal 13. Indeed, the topic is a good one. However, authors need to address the following issues:

Major issues

1. The authors failed to explain how their topic is important in the background.
2. the methodology lacks some clarity; how data will be reported eg. Authors can propose and adopt the PRISMA flow diagram to report data.

Minor issues

1. authors failed to prof-read the manuscript and that the manuscript contains unacceptable typographical errors and grammatical mistakes e.g.

Please check the lines below from the background of the manuscript:

63	comprises of – comprise does not take a preposition

67	CC are as a result of increase – subject and verb agreement (as a result of increases)

69	were link – subject and verb agreement (were linked)

71	effects on - wrong use of preposition. It should be the effects ‘of’

73	double negative – not and not

78	utilised to applied – to should be followed by a verb, so to apply

83	benefit of all parties - incorrect preposition (benefit for all parties)

84	Dialogues involves - wrong use of verb (it should be ‘involve’)

These mistakes are not exhaustive. All other sections are full of errors and mistakes.

**Suggestion:**

I would like to suggest that:

1. the authors proofread the manuscript and correct all the avoidable errors.
2. Pretest their questions and if necessary amen the topic per the existing literature
3. Read on the PRISMA flow diagram and capture it in this protocol
