# Supplementary material for: A planetary health perspective on the translation of climate change research into public health policy and practice: A scoping review protocol
Source: PLoS One. 2024 Mar 13;19(3):e0295931. doi: 10.1371/journal.pone.0295931 (PMC10936763; doi:10.1371/journal.pone.0295931)
Supplement: Attachment — Submitted filename: Response to Reviewers_ Rebuttal Table_KTCC_26_11_23.docx [file pone.0295931.s007.docx]

Rebuttal Table

Manuscript title: A planetary health perspective on translation of climate change research into public health policy and practice: A scoping review protocol

| **Reviewer 1** |  |  |
| --- | --- | --- |
| Comments | Response to Comments | Changes made in the document |
| Pg. 4: “In 2019 it was estimated that 356, 000 deaths worldwide were link to extreme heat (7),…..”   (i) Change ‘link’ to linked | The authors have changed the word link to “linked” | Page 6 line 112 |
| “In 2019 it was estimated that 356, 000 deaths worldwide were link to extreme heat (7),…..”   (i) Not all extreme heat could be attributed to CC. The authors need to situate this statement appropriately in the context of CC | The authors have substantiated the sentence and situated in the context of climate change. | Page 4 line 68-74 |
| “In literature, several have been conducted to establish effects on CC………”   (i) Several studies?? | The authors have included the word “studies” | Page 4 line 75 |
| “However, CC research knowledge has not been not translated into tangible solution in relation to public health policy and practice”.   (i) Kindly delete ‘not’ before translated | The authors have removed the second “not” |  |
| “Integrated knowledge translation (IKT) approach is one strategy that can be utilised to applied”….   (i) To apply?? | The authors have removed the word “applied” |  |
| There are places climate change is shortened as “CC” and in some places written fully. I think this should be consistent throughout the paper | The authors have made the changes in the document and made it consistent by using “CC” throughout the document. | Changes made throughout the document. |
| Pg.5  ‘we have not identified any reviews on decision making in climate change……   (i) We have not identified any review……please delete the ‘s’ | The authors have deleted the “s” |  |
| Page 8  “Any studies…….”   (i) Rephrase to Any study | We have rephrased to any review | Page 6. Line 112-113 |
| Type of participants  This section does not come clear to readers. This is a scoping review so mentioning study participants such as researchers working in the field of climate change etc does come to clear. Maybe authors need to explain this section and the role of those participants in the scoping review. | We have revised this section and provided clarity on what we mean by participants by saying that we are interested in identifying the participants or stakeholders mentioned in the articles | Pg. 9-10 Line 202-212 |
| Are the authors using the PCC framework in this regard? More explanation needed or that whole section could be deleted | We have included the PCC framework will be applied to the scoping review. | Pg. 7 Line 141-143 |
| Can the authors expand the database to Scopus etc. It appears what has been listed is too limiting. | We have decided to expand the database to include Scopus | Page 8 line 169 |
| Again, will the study include only English Language papers? That needs to be stated clearly | We had indicated previously that only studies conducted in English will be included in the study. | Page 8 line 170-171 |
| Will the entire scoping review be guided by a theoretical framework on CC and Health? If so, this must be stated in the manuscript. | We will draw on the planetary health framework where appropriate to guide data extraction, analysis and synthesis. |  |
| Overall, I think the authors need to read through the manuscript again and correct some basic grammatical errors which could have been avoided. | The authors have had the manuscript proof read. |  |
| **Reviewer2** |  |  |
| The significance, rationale and objectives are justified. | Thank you, we appreciate the positive feedback. |  |
| -The background – page 4 line 15 ‘Many deaths have been attributed to CC are as a result of increase in temperatures, and *changing patterns of infectious diseases among vulnerable communities* (6). If necessary explain the link between CC and changing patterns of infectious disease among vulnerable groups.  Are there other CC related factors responsible for deaths? | Thank you for the comment. While it may be an interesting prospect to discuss the link between CC and how it affects infectious diseases mechanism, it is beyond the scope of this review and outside our defined objectives. However, we have added a sentence in line with your suggestion.  A few more factors have been included. Thank you | Pg. 4 line 63-68 |
| Include some literature on research knowledge translation. | We have included literature on knowledge translation research. | Pg. 5 Line 92-104 |
| Need some editing. | Thank you for the comment the article has been edited. |  |
| -It is commendable that authors will be guided by Arksey and O’Malley methodology. However, this methodology though offers a good foundation, authors should highlight strengths and potential limitations that are inherent in this methodology. | Thank you, we have included the strengths and limitations of conducting a scoping review highlighted by Arksey and O’Malley. | Pg.7 Line 149-153 |
| Identifying relevant studies: Review time is unclear: 2000 onwards (2000-2022) or last two decades. Please clarify. | We have rectified and stated that we will search for studies from 2003-2023 | Pg. 8 Line 159 |
| The preliminary search strategy using relevant keywords and MeSH terms (Supplementary File 2), I suggest to add AND NOT to Boolean Operators OR and AND. | WE have revised the search string and added AND NOT to the Boolean Operators OR and AND. | The search output has been updated (see Supplementary File 2) |
| Clarify who is conducting the search- the authors, author reviewers, or reviewers. The study will benefit from a librarian? | I have clarified that I (CM)the main author will conduct the initial searches with the assistance of a librarian  Additionally, the search of the reference list of included studies and contacting authors will be done be myself (CM) and the second author (SA) | Pg. 8 Line 162  Pg. 9 Line 187 |
| Covidence: Please clarify how Covidence will be used in managing this scoping review? | We have clarified in the manuscript that Covidence will be used to screen, extract data and keep track of the screening. | Pg. 9 Line 194-197 |
| Collating summarizing, and reporting the results: explain more on qualitative analysis process. | We have stated that we will apply the six steps of thematic analysis by Braun and Clark. | Pg. 11 Line 237-240 |
| **Reviewer 3** |  |  |
| Pre-testing is necessary for scoping reviews to ensure whether there is existing literature on the research topic. However, the authors failed to produce evidence that their study is feasible. | Thank you for your comment. We have provided proof that our study is feasible. | Kindly see supplementary File 2 that shows that the search string in PubMed yielded 1879 articles |
| Major issue: 1. The authors failed to explain how their topic is important in the background. | The authors have added more text to justify why the study is important and why it should be conducted. | Pg. 6 line 116-129 |
| Major issue: 2. the methodology lacks some clarity; how data will be reported eg. Authors can propose and adopt the PRISMA flow diagram to report data. | Thank you for the comment it was mentioned previously that the PRISMA flow diagram will be used to report the data. | Pg. 8 Line 154 |
| Minor issues: 1. authors failed to prof-read the manuscript and that the manuscript contains unacceptable typographical errors and grammatical mistakes e.g. Please check the lines below from the background of the manuscript: | Thank you for the comment. The authors have proof-read the entire manuscript. |  |
| 63. comprises of – comprise does not take a preposition | Thank you, we have removed “of” |  |
| 67. CC are as a result of increase – subject and verb agreement (as a result of increases) | Thank you we have edited this from increase to increases. | Pg. 4 line 64 |
| 69. were link – subject and verb agreement (were linked) | Thank you, we have worked on this and changed the term from link to linked. | Pg. 4 line 70 |
| 71. effects on - wrong use of preposition. It should be the effects ‘of’ | We have edited accordingly | Pg. 4 line 75 |
| 73. double negative – not and not | We have edited this as per your request. |  |
| 78. utilised to applied – to should be followed by a verb, so to apply | We have edited this has per your suggestion | Pg. 5 line 81-82. |
| 83. benefit of all parties - incorrect preposition (benefit for all parties) | We have edited this as per your suggestion | Pg. 5 line 86 |
| 84. Dialogues involves - wrong use of verb (it should be ‘involve’) | We have edited this as per your request | Pg. 5 line 87 |
| These mistakes are not exhaustive. All other sections are full of errors and mistakes. | We took note of this and proofread the article. |  |
| The authors proofread the manuscript and correct all the avoidable errors. | We have done this thank you |  |
| Pretest their questions and if necessary amend the topic per the existing literature | We have pre-tested and conducted the search for study feasibility. Kindly see supplementary File 2. Thank you |  |
| Read on the PRISMA flow diagram and capture it in this protocol | We have done this. Thank you. |  |
